# Census Tract–Level Physical and Social Environmental Determinants of Edentulism: A Machine Learning Approach

**DOI:** 10.1093/geroni/igaf122.1023

**Published:** 2025-12-31

**Authors:** Xiang Qi, Jie Yao, Bei Wu, Shaoyong Su, Xiaoling Wang, Kai Zhang

**Affiliations:** New York University, New York, New York, United States; University at Albany School of Public Health, Hampton Manor, New York, United States; NYU Shanghai, Shanghai, China (People’s Republic); Augusta University, Medical College of Georgia, Augusta, Georgia, United States; Augusta University, Medical College of Georgia, Augusta, Georgia, United States; UNT Health, Fort Worth, Texas, United States

## Abstract

Edentulism (i.e., complete tooth loss) disproportionately affects older US adults in marginalized communities, driven by complex social and environmental factors. There is a critical gap in understanding how these factors interact at the population level to shape oral health disparities. This study employed Extreme Gradient Boosting (XGBoost), a machine learning technique, to analyze these determinants across 38,379 census tracts using 2021 data. Social environmental determinants—such as poverty rates, education levels, and employment status—were sourced from the American Community Survey and aggregated at the census tract level to capture localized socioeconomic conditions. Physical environmental determinants, including climate zone and access to dental care, were similarly aggregated to reflect tangible surroundings impacting health. The model predicted edentulism rates among adults aged 65+ with high precision, explaining 87% of the variance. Social vulnerability and climate zone emerged as the top predictors, underscoring the interplay between socioeconomic disadvantage and geographic conditions. Census tract characteristics, like poverty and racial segregation, moderated these effects, revealing distinct risk profiles—e.g., in high-poverty tracts, social factors amplified edentulism risk differently than in racially segregated ones. These findings highlight the contextual nature of oral health and demonstrate the value of localized, data-driven analyses. By identifying key social and physical determinants, this research informs targeted interventions to reduce oral health disparities. The use of XGBoost showcases machine learning’s potential to dissect complex health issues, offering actionable insights for policymakers and clinicians to improve outcomes in diverse settings.

